# Retinal Thickness Analysis in Progressive Multiple Sclerosis Patients Treated With Epigallocatechin Gallate: Optical Coherence Tomography Results From the SUPREMES Study

**DOI:** 10.3389/fneur.2021.615790

**Published:** 2021-04-28

**Authors:** Katharina Klumbies, Rebekka Rust, Jan Dörr, Frank Konietschke, Friedemann Paul, Judith Bellmann-Strobl, Alexander U. Brandt, Hanna G. Zimmermann

**Affiliations:** ^1^Experimental and Clinical Research Center, Max Delbrueck Center for Molecular Medicine and Charité—Universitätsmedizin Berlin, Corporate Member of Freie Universität Berlin and Humboldt-Universität zu Berlin, Berlin, Germany; ^2^NeuroCure Clinical Research Center, Charité—Universitätsmedizin Berlin, Corporate Member of Freie Universität Berlin and Humboldt-Universität zu Berlin, Berlin, Germany; ^3^Neurology Department, Oberhavel Clinic, Hennigsdorf, Germany; ^4^Institute of Biometry and Clinical Epidemiology, Charité—Universitätsmedizin Berlin, Corporate Member of Freie Universität Berlin and Humboldt-Universität zu Berlin, Berlin, Germany; ^5^Department of Neurology, Charité—Universitätsmedizin Berlin, Corporate Member of Freie Universität Berlin and Humboldt-Universität zu Berlin, Berlin, Germany; ^6^Department of Neurology, University of California, Irvine, Irvine, CA, United States

**Keywords:** optical coherence tomography, retina, progressive multiple sclerosis, treatment response, epigallocatechin gallate

## Abstract

**Background:** Epigallocatechin gallate (EGCG) is an anti-inflammatory agent and has proven neuroprotective properties in animal models of multiple sclerosis (MS). Optical coherence tomography (OCT) assessed retinal thickness analysis can reflect treatment responses in MS.

**Objective:** To analyze the influence of EGCG treatment on retinal thickness analysis as secondary and exploratory outcomes of the randomized controlled *Sunphenon in Progressive Forms of MS* trial (SUPREMES, NCT00799890).

**Methods:** SUPREMES patients underwent OCT with the Heidelberg Spectralis device at a subset of visits. We determined peripapillary retinal nerve fiber layer (pRNFL) thickness from a 12° ring scan around the optic nerve head and thickness of the ganglion cell/inner plexiform layer (GCIP) and inner nuclear layer (INL) within a 6 mm diameter grid centered on the fovea from a macular volume scan. Longitudinal OCT data were available for exploratory analysis from 31 SUPREMES participants (12/19 primary/secondary progressive MS (PPMS/SPMS); mean age 51 ± 7 years; 12 female; mean time since disease onset 16 ± 11 years). We tested the null hypothesis of no treatment^*^time interaction using nonparametric analysis of longitudinal data in factorial experiments.

**Results:** After 2 years, there were no significant differences in longitudinal retinal thickness changes between EGCG treated and placebo arms in any OCT parameter (Mean change [confidence interval] ECGC vs. Placebo: pRNFL: −0.83 [1.29] μm vs. −0.64 [1.56] μm, *p* = 0.156; GCIP: −0.67 [0.67] μm vs. −0.14 [0.47] μm, *p* = 0.476; INL: −0.06 [0.58] μm vs. 0.22 [0.41] μm, *p* = 0.455).

**Conclusion:** Retinal thickness analysis did not reveal a neuroprotective effect of EGCG. While this is in line with the results of the main SUPREMES trial, our study was probably underpowered to detect an effect.

**Clinical Trial Registration:** www.ClinicalTrials.gov, identifier: NCT00799890.

## Introduction

Multiple sclerosis (MS) is the most common autoimmune inflammatory and degenerative central nervous system (CNS) disease, often resulting in sustained neurological deficits (1). The majority of patients manifest with a relapsing remitting (RRMS) disease course (2, 3), followed by a secondary progressive (SPMS) stage ~20 years from onset (4). However, 15–20% show a primary progressive (PPMS) disease course from onset (3, 5, 6). Neurodegeneration may be present in any course from the onset of the disease (7–10).

The principle of disease modifying therapy (DMT) aims at decreasing relapse frequency and disability progression. Whereas various immunomodulatory drugs for the treatment of RRMS targeting the inflammatory aspect of the disease have been established in the last decades (11), treatment options for progressive MS are sparse (12, 13). Furthermore, due to the absence of clinical relapses, treatment response is difficult to measure in progressive MS and has to rely on measures not primarily associated with relapse activity (13).

Green tea anti-inflammatory, anti-oxidative, and anti-cancerogenic effects have been shown on various conditions such as energy metabolism, cell development, and neuroprotection (14–17). The most active agent is the polyphenol epigallocatechin-gallate (EGCG), comprising 50–80% of the total catechins in green tea (18). EGCG has shown immunomodulatory effects by inhibition of T cell proliferation and thus modulates the production of T cell-derived cytokines, e.g., Interferon-γ, Interleukin-2, and tumor necrosis factor (TFN) α (from T helper type 1 cell subset) (19–21). In an experimental animal model of MS (experimental autoimmune encephalomyelitis, EAE) the oral intake of EGCG suppressed inflammation via inhibition of TNFα and nuclear factor kappa-light-chain-enhancer of activated B cells in T cells, thus resulting in reduced clinical disease severity and fewer CNS lesions in mice (22–24). Furthermore, treatment with EGCG and glatiramer acetate in EAE mice delayed disease onset, reduced clinical disability and reduced inflammatory infiltrates (25). In clinical trials, oral intake of EGCG was associated with improved muscle metabolism during moderate exercise in RRMS (26) and improved cognitive rehabilitation in genetic disorders (27, 28).

Optical coherence tomography (OCT) allows quantification of anterior visual pathway damage in MS patients (29–33). While thinning of the peripapillary retinal nerve fiber layer (pRNFL), containing unmyelinated axons, and the ganglion cell layer, containing their cell bodies, reflect neuroaxonal atrophy as a consequence of retrograde neurodegeneration, the inner nuclear layer (INL) is associated with inflammation manifesting in thickening and edema (31, 34–40). The ganglion cell layer is usually—due to similar contrast on OCT images—analyzed in combination with the inner plexiform layer (GCIP). RNFL and GCIP changes are found even during early stages of MS and occur also in absence of a history of optic neuritis (ON) (8, 41–44). Response to DMT is reflected by decreased rates of GCIP thinning (45) and thinning of INL in RRMS patients (46). A recent study has shown faster retinal thinning—also compared to RRMS patients and no effect of DMT on thinning rates in progressive MS (47). The study has been discussed controversially (48).

The SUPREMES study (Sunphenon in progressive forms of multiple sclerosis) was a phase 2 monocentric, prospective, randomized double-blind placebo-controlled pilot study to evaluate the effect of EGCG/Sunphenon on brain atrophy in MRI over a period of 36 months in patients with primary and secondary progressive multiple sclerosis (NCT00799890). The primary results of the SUPREMES study have been published elsewhere (49). OCT parameters were assessed as secondary and exploratory outcomes. The aim of our study was to evaluate the impact of EGCG on longitudinal retinal component changes in patients with progressive MS.

## Materials and Methods

### Patients and Study Design

In total, 61 patients were randomized to the SUPREMES trial (NCT00799890) at the NeuroCure Clinical Research Center (NCRC) at Charité—Universitätsmedizin Berlin, Germany. Inclusion and exclusion criteria, randomization, blinding process and primary and secondary endpoints are described in detail elsewhere (49). Primary outcome parameter of the main study was brain atrophy detected as the difference between brain parenchymal fraction after 36 months compared to baseline. Inclusion criteria were age between 18 and 65 years, diagnosis of primary progressive or secondary progressive multiple sclerosis according to the McDonald criteria version 2005 (50), expanded disability status scale (EDSS) (51) between 3.0 and 8.0 and at least 30 days between the last exacerbation and study screening. Exclusion criteria were treatment with any immunomodulatory or immunosuppressive drugs, with exception of methylprednisolone up to 3 months before screening. Regarding OCT, pRNFL was a secondary outcome parameter; GCIP and INL were analyzed as exploratory endpoints. For inclusion in the analysis of OCT, ophthalmological diseases such as glaucoma, recurrent iritis, myopia <-5 dpt were considered as additional exclusion criteria. As for many patients OCT scanning was not available in the beginning, we only included patients to the OCT analysis who had at least one follow-up OCT at least 6 months from baseline OCT.

### Study Medication

Patients in the treatment arm started treatment with one capsule containing Sunphenon 200 mg/day and placebo patients received identical capsules without active component. After 3 months, participants received two capsules per day of either EGCG or placebo medication. After 6 months, the medication increased to 600 mg/day, after 18 months to 800 mg/day and after 30 months they received the full amount of 1,200 mg/day.

### Ethics

The SUPREMES trial was approved by the local ethics committee (LaGeSo ZS EK 10 407/08, new: 08/0407-EK 15) and by the German Federal Institute for Drugs and Medical Devices (BfArM). The trial is registered with EudraCT (2008-005213-22) and clinicaltrials.gov (NCT00799890) and was conducted in accordance with the current version of the Declaration of Helsinki and the applicable German law. All subjects provided written informed consent prior to enrolment.

### Optical Coherence Tomography

Patients underwent spectral domain OCT (Spectralis SD-OCT; Heidelberg Engineering, Heidelberg, Germany) with the Eye Explorer 1.9.10.0 and automatic real-time (ART) image averaging. pRNFL was calculated from a standard ring scan around the optic nerve head (12°, 1536 A-scans, 16 ≤ ART ≤ 100) using segmentation by the device's software with viewing module 6.0.14.0. A macular volume scan (25° × 30°, 61 B-scans, 768 A-scans per B-scan, 12 ≤ ART ≤ 15) was acquired for intraretinal segmentation of GCIP and INL. Segmentation of macular scans was performed with SAMIRIX (52). All OCT scans were revised for retinal changes unrelated to MS, sufficient quality (53, 54), segmentation errors and were manually corrected by a blinded experienced grader if necessary. OCT methods are reported in line with the APOSTEL criteria (55).

### Statistical Methods

**Figure 1:**
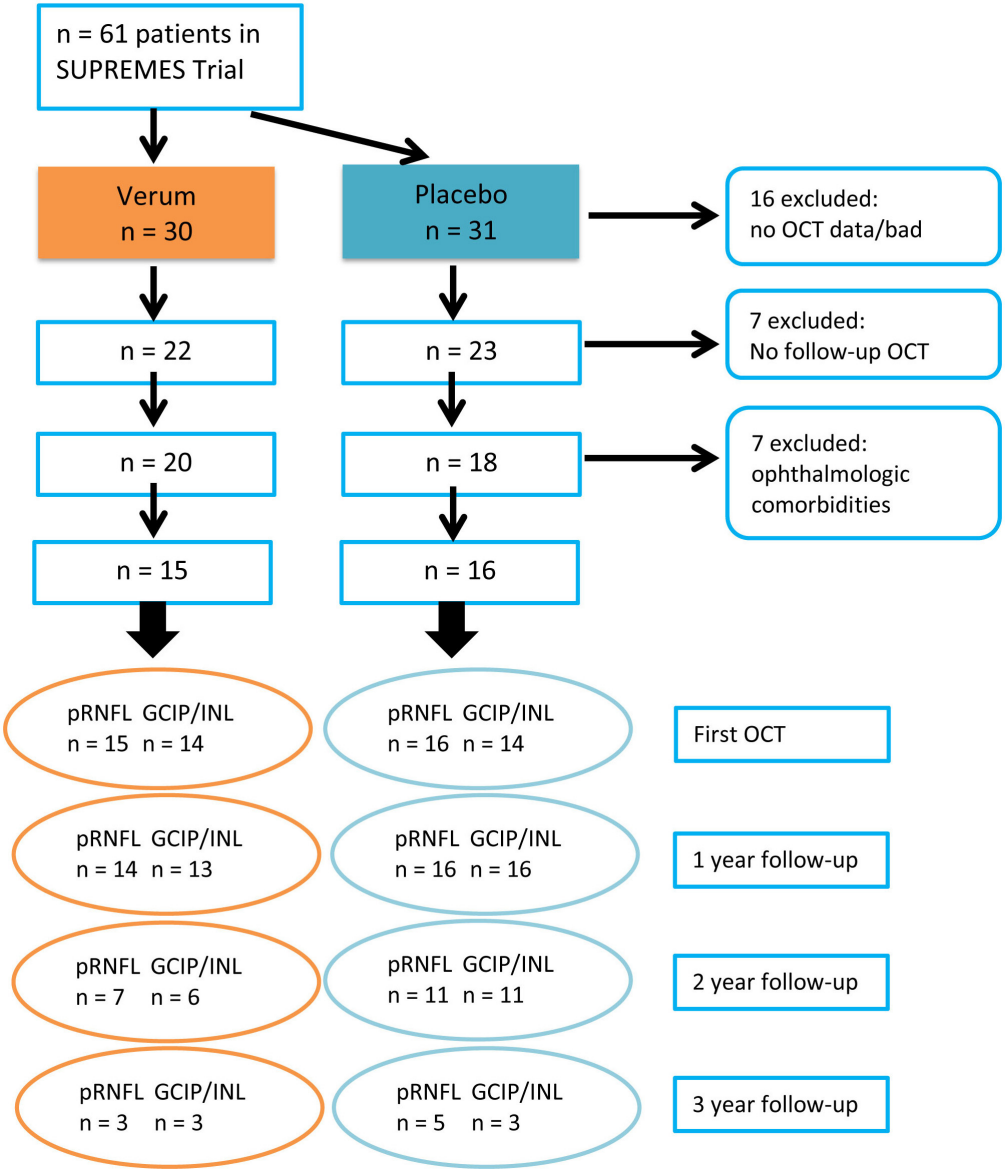
CONSORT chart describing the enrolment process of OCT analysis and case numbers at each year of follow-up. PMS, progressive MS; OCT, Optical coherence tomography; pRNFL, peripapillary retinal nerve fiber layer; GCIP, ganglion cell and inner plexiform layer; INL, inner nuclear layer.

Cohort baseline differences with subject reference in numerical variables were either given as mean ± standard deviation and analyzed with *t*-test, or as median and interquartile range (IQR) and analyzed with Wilcoxon rank-sum test, while Chi-Square test was applied for categorical variables. Due to overall low sample size and high number of missing data (Figure 1) we tested the OCT first examination and the longitudinal main hypothesis with “nonparametric analysis of longitudinal data in factorial experiments” as implemented in the R package nparLD (56). We modeled first OCT examination within an F1-LD-F1 design and used the ANOVA-Type test with treatment arm as whole-plot factor and eye as sub-plot factor for inference. We performed longitudinal analysis within the F1-LD-F2 experimental design with one whole-plot factor and two sub-plot factors, where the second sub-plot factor is the stratification of the first. Using this design, we used treatment group as whole-plot factor, time as the first subplot factor, and eye as the second to account for two eye measurements per patient at each time point. We excluded three-year follow up because of potential bias resulting from missing data. The main question was whether the time profiles of the two groups were parallel or diverging, i.e., if there exists a statistical interaction between treatment group and time after 2 year follow up, which would indicate an effect of EGCG on OCT changes over time. The effect size is represented by the relative marginal treatment effect (RTE), indicating whether data tend to be smaller/larger under respective factor level combinations. The analysis set included missing values as described in the flow chart (Figure 1). In this data set we rounded follow-up time to full years in order to use time as a categorical variable. To confirm our findings, changes in OCT parameters were estimated with linear mixed models (LMM) using the formula: OCT value ~ group^*^time from baseline + (1 + time from baseline|patient/eye). In LMM, all sessions were considered including time since baseline as a continuous variable. No corrections for multiple comparisons were performed for this exploratory outcome analysis. Statistical analyses were performed with R (57) version 3.6.2 with packages nparLD (56), lme4, lmertest, tidyverse, tableone, ggplot2, beeswarm, ggplot, RMisc. Statistical significance was established at *p* < 0.05.

## Results

### Cohort Description

Sixty-one patients with progressive MS were randomized in the SUPREMES trial to receive either EGCG treatment or placebo. From these patients, we had to exclude 16 patients because of missing OCT data. From the 45 patients with OCT data, seven patients had no follow-up OCT data, and 7 patients had to be excluded due to ophthalmological diseases such as glaucoma, recurrent iritis, and myopia <-5 dpt. Thus, 31 patients were included in analysis. The inclusion process is detailed in Figure 1. Moreover, from 2 patients (1 EGCG, 1 placebo), one eye was excluded from all analyses because of unilateral retinopathy. Two pRNFL scans from 2 patients (both EGCG) and 34 macular scans from 28 sessions of 20 patients (8 EGCG, 12 placebo) failed the OSCAR-IB quality criteria and had to be excluded (53, 54).

### Baseline OCT Findings

Baseline cohort details are described in Table 1. Patients had their first OCT examination median 1.05 (interquartile range 0.00–1.52) years after randomization. The OCT cohort comprised 15 patients from the treatment and 16 patients from the placebo group. There were no significant differences in age, sex, time since disease onset, EDSS, time in the trial, and follow-up duration between treatment and placebo groups (Table 1). Patients in the EGCG treated arm had thicker GCIP, INL, and—though not significant—pRNFL (Table 2).

**Table 1:** Baseline cohort description.

		**EGCG**	**Placebo**	***p***
***n***		**15**	**16**	
**Age [years]**		50.8 ± 8.4	50.7 ± 6.9	0.968
**Sex female [*****n*** **(%)]**		5 (33.3)	7 (43.8)	0.821
**Diagnosis**	**PPMS [*****n*** **(%)]**	6 (40.0)	6 (37.5)	>0.999
	**SPMS [*****n*** **(%)]**	9 (60.0)	10 (62.5)	
**Disease duration [years] (median, [IQR])**		13.69 [8.90, 29.41]	12.12 [7.47, 20.17]	0.406
**EDSS (median, IQR)**		6.00 [4.00, 6.50]	5.75 [4.00, 6.00]	0.138
**Time on trial at OCT baseline (median, IQR) [years]**		1.06 [0.00, 1.50]	1.04 [0.00, 1.53]	0.919
**Follow-up duration (median, IQR) [years]**		1.47 [1.27, 2.01]	1.95 [1.47, 2.90]	0.213

*Abbreviations: EGCG: epigallocatechin-gallate, SPMS: secondary progressive multiple sclerosis, PPMS: primary progressive multiple sclerosis, EDSS: Expanded disability status scale, IQR: interquartile range, OCT: optical coherence tomography*.

**Table 2:** First OCT measurements.

	**EGCG**		**Placebo**		**EGCG vs. placebo**
	**Mean ± SD**	**RTE**	**Mean ± SD**	**RTE**	***p***
pRNFL/μm	87.3 ± 11.1	0.554	82.9 ± 11.4	0.450	0.297
GCIP/μm	65.4 ± 7.4	0.609	59.9 ± 6.1	0.381	0.024
INL/μm	37.8 ± 2.2	0.599	36.1 ± 2.3	0.392	0.049

*Test statistics from “nonparametric analysis of longitudinal data” of first examination OCT data. EGCG, epigallocatechin-gallate; CI, confidence interval; RTE, Relative treatment effect; pRNFL, peripapillary retinal nerve fiber layer; GCIP, ganglion cell and inner plexiform layer; INL, inner nuclear layer*.

### Longitudinal OCT Results

**Figure 2:**
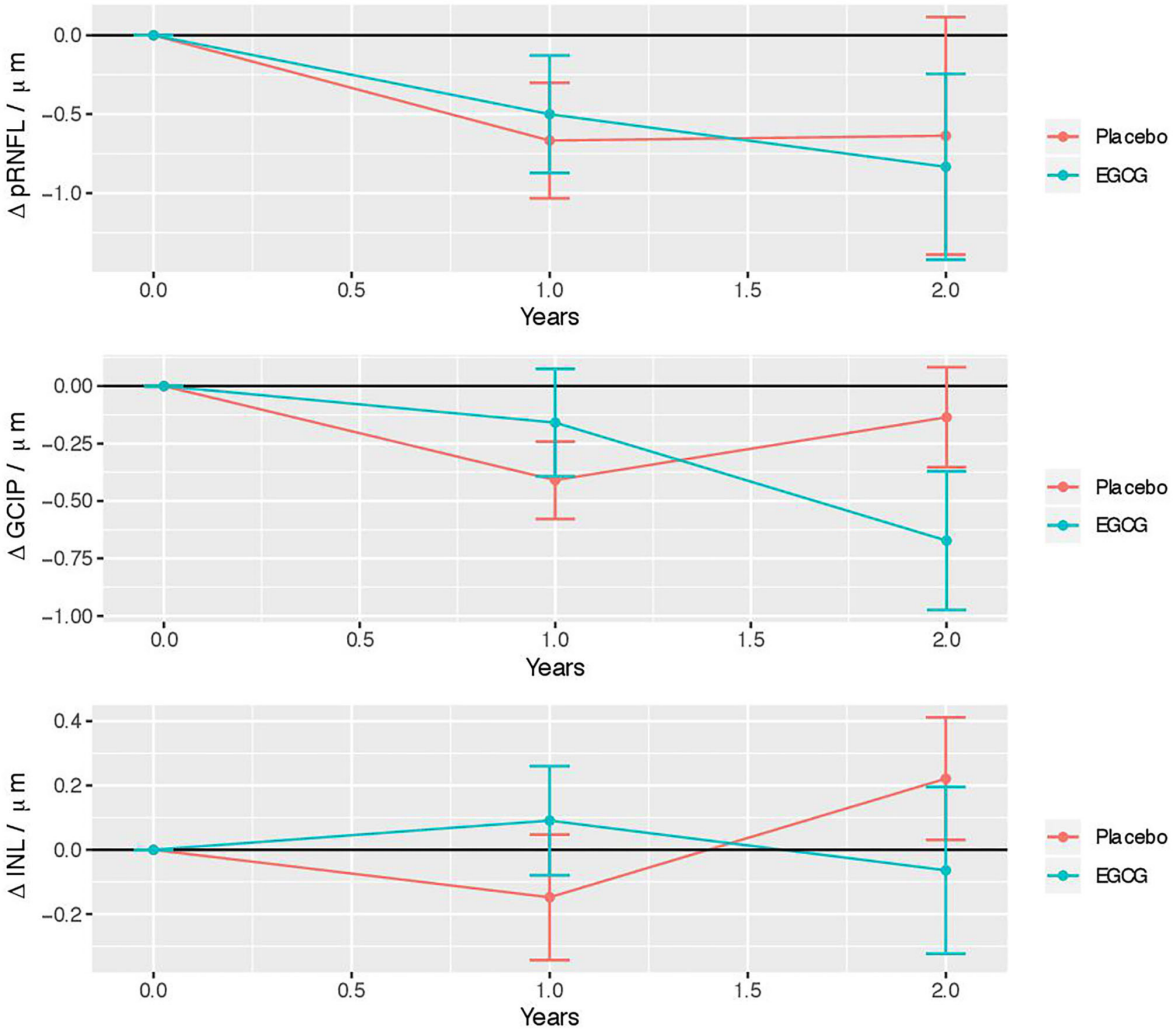
Longitudinal retinal layer changes in the EGCG treated and placebo group. Error bars indicate the standard error to the mean. EGCG, epigallocatechin-gallate; pRNFL, peripapillary retinal nerve fiber layer; GCIP, ganglion cell and inner plexiform layer; INL, inner nuclear layer.

Figure 2 illustrates changes over time in the EGCG treated and Placebo group. Table 3 depicts changes over time separately for the treatment and the placebo arms and their statistical comparison from non-parametric longitudinal data analysis. There was no significant interaction of treatment and time for any parameter. Table 4 includes results from LMMs, as well not detecting any significant differences in change over time between ECGC and placebo group.

**Table 3:** Longitudinal OCT changes in treatment arms—nonparametric analysis.

	**EGCG**		**Placebo**		**EGCG vs. Placebo**
	**Mean change [CI]/μm**	**RTE**	**Mean change [CI]/μm**	**RTE**	***p***
		**treatment:time**		**treatment:time**	
pRNFL/μm	−0.83 [1.29]	0.331	−0.64 [1.56]	0.492	0.156
GCIP/μm	−0.67 [0.67]	0.360	−0.14 [0.47]	0.429	0.476
INL/μm	−0.06 [0.58]	0.504	0.22 [0.41]	0.635	0.455

*All results for the 2-year follow-up visit. Test statistics from “nonparametric analysis of longitudinal data.” EGCG, epigallocatechin-gallate; RTE, Relative treatment effect; pRNFL, peripapillary retinal nerve fiber layer; GCIP, ganglion cell and inner plexiform layer; INL, inner nuclear layer*.

**Table 4:** Longitudinal OCT changes in treatment arms—linear mixed models.

		***B***	**SE**	***p***	**Lower CI**	**Upper CI**	***R*^**2**^m**	***R*^**2**^c**
pRNFL	Treatment EGCG	3.194	4.014	0.433	−4.673	11.062	0.032	0.982
	Time	−0.788	0.306	0.018	−1.387	−0.189		
	Treatment EGCG:Time	0.766	0.463	0.111	−0.140	1.673		
GCIP	Treatment EGCG	4.389	2.432	0.082	−0.379	9.156	0.092	0.994
	Time	−0.221	0.111	0.068	−0.439	0.003		
	Treatment EGCG:Time	0.0138	0.160	0.933	−0.300	0.327		
INL	Treatment EGCG	1.866	0.838	0.034	0.223	3.509	0.136	0.956
	Time	−0.075	0.084	0.374	−0.240	0.089		
	Treatment EGCG:Time	0.064	0.119	0.589	−0.168	0.297		

*All result for the maximum available follow-up time (continuous) under treatment. Test statistics from linear mixed models. B, non-standardized correlation coefficient; SE, standard error; CI, 95% confidence interval; R^2^m, Marginal R^2^; R^2^c, Conditional R^2^; pRNFL, peripapillary retinal nerve fiber layer; GCIP, ganglion cell and inner plexiform layer; INL, inner nuclear layer*.

## Discussion

In this study, we performed an analysis of OCT data as secondary (pRNFL) and exploratory (GCIP, INL) outcomes in the SUPREMES trial. Specifically, we investigated differences in retinal thickness changes over time between patients treated with EGCG vs. placebo. We found no difference between the treatment groups.

These results support the findings in the analysis of the primary and secondary outcome parameters of the SUPREMES trial: no evidence for treatment was found on brain atrophy, lesion load, and clinical scores (49). The primary outcome parameter of the SUPREMES trial was brain atrophy, a commonly used outcome for neuroprotective trials in MS (58). While brain atrophy measurement is widely established, retinal thickness analysis has been included as an additional outcome as the use of brain atrophy is not without challenge: a reduction of acute swelling by a potent anti-inflammatory intervention may lead to the phenomenon of “pseudoatrophy,” which is referred to as decreased brain volume due to the resolution of edema and inflammation after treatment (59, 60). Furthermore, as our cohort had an average age of 50 years, treatment effects on brain atrophy may be confounded by non-linear aging effects (61).

In contrast, retinal thickness measurements are less prone to aging (52). Furthermore, GCIP is not prone to swelling (62), whereas a subtle swelling of pRNFL outside of acute ON has not been reported so far. While they may be inferior to brain atrophy at face value, GCIP and pRNFL may be superior for detecting neuroprotective effects due to a lack of pseudoatrophy. Nevertheless, we did not find a significantly reduced atrophy of pRNFL and GCIP in the EGCG group.

While pRNFL and GCIP thinning reflect neuroaxonal damage, the INL is considered a marker of inflammation. Treatment response is considered to be associated with INL thinning (46). However, the INL is also subject to atrophy as indicated by thinning in a large progressive MS study (47). In our study, the INL showed no overall thickness changes. This suggests that either no time-dependent change occur, or that both atrophy and inflammation occur in our cohort, masking a treatment-associated thinning.

Other clinical trials also failed to show a treatment effect of EGCG: The SUNIMS trial (63) reported no treatment effect of EGCG on clinical or MRI measures in RRMS patients. Moreover, a recently published study demonstrated no impact of EGCG after 48 weeks of treatment on disease progression in multiple system atrophy (64). A potential reason for the failure of EGCG in clinical trials could be the lower bioavailability of oral EGCG than previously assumed (65, 66).

Several limitations may impact our results. First, the low sample size of our cohort. A previous study estimated that the sample size for a progressive MS trial on neuroprotective agents should be at least *n* = 173 for pRNFL and *n* = 125 for GCIP per trial arm for a 3-year study (power 80%, effect size 50%), numbers way larger than achieved in this exploratory outcome analysis (47).

Another weakness is that treatment and placebo groups were not well-matched regarding baseline OCT, with a significantly thicker GCIP and INL in the treatment group. In our non-parametric analysis, we used the change of retinal parameters as outcome and the linear mixed models we computed additionally consider the individual intercept at baseline. Thus, we assume that the differences at OCT baseline had no influence on the longitudinal analysis.

To date, there are few studies applying OCT as an outcome parameter in clinical trials of MS. To the best of our knowledge, there is no published prospective interventional study that applied OCT as outcome parameter in trials in the progressive forms of the disease. While OCT detected differences in retinal thickness change between different treatment groups in RRMS (45), it is possible that the retina of SPMS and PPMS patients are less responsive to treatment. Another aspect is the high frequency of primary eye disorders in a usually elder progressive MS population. In our study, almost 20% of patients needed to be excluded due to eye comorbidities. Furthermore, due to increased disability, progressive MS patients are often less compliant with the OCT examination, leading to a high number of noise or cut-off scans failing the quality control. While this does not preclude OCT as endpoint from clinical trials in progressive MS, it suggests that careful ophthalmological examination for comorbidities and rigorous quality control of OCT scans are of paramount importance. A recent retrospective study showed a decreased macular RNFL thinning associated with 4-aminopyridine treatment in a mixed cohort of RRMS and progressive MS patients (67). These and our results encourage the further evaluation of OCT measurements as outcome parameters in clinical trials of progressive MS.

To conclude, our study shows no effect over time of EGCG on pRNFL, GCIP, or INL. As such, our study does not provide sufficient evidence for a neuroprotective effect of EGCG on retinal thickness in patients with SPMS and PPMS. While this is in line with the outcomes of the main SUPREMES trial, our study was probably underpowered to detect a treatment effect.

## Data Availability Statement

The raw data supporting the conclusions of this article will be made available upon request to the corresponding author to any qualified researcher.

## Ethics Statement

The studies involving human participants were reviewed and approved by The SUPREMES trial was approved by the local ethics committee (LaGeSo ZS EK 10 407/08, new: 08/0407-EK 15) and by the German Federal Institute for Drugs and Medical Devices (BfArM). The trial is registered with EudraCT (2008-005213-22) and clinicaltrials.gov (NCT00799890). The patients/participants provided their written informed consent to participate in this study. Written informed consent was obtained from the individual(s) for the publication of any potentially identifiable images or data included in this article.

## Author Contributions

KK drafting/revising the manuscript, analyzed and interpreted the data, and acquisition of data. RR acquisition of data, interpreted the data, and revised the manuscript for intellectual content. JD, FP, and JB-S study concept, acquisition of data, and revised the manuscript for intellectual content. FK analyzed and interpreted the data, statistical analysis, and revised the manuscript for intellectual content. AB study concept, analyzed and interpreted the data, statistical analysis, and drafting/revising the manuscript. HZ study concept, acquisition of data, analyzed and interpreted the data, statistical analysis, and drafting/revised the manuscript for intellectual content. All authors contributed to the article and approved the submitted version.

## Conflict of Interest

RR received speaking honoraria from Roche. JD reports research support by Bayer and Novartis, honoraria for lectures and advisory by Bayer, Novartis, Sanofi-Aventis, Merck-Serono, Biogen and Roche and travel support by Bayer, Novartis, Biogen, and Merck-Serono. FP reports non-financial support from Taiyo International, grants from TEVA GmbH, other from German Research Council (DFG), during the conduct of the study; He serves on scientific advisory boards of Novartis's OCTIMS study steering committee and MedImmune/Viela Bio steering committee. He received funding for travel or speaker honoraria from Bayer, Novartis, Biogen Idec, Teva, Sanofi-Aventis/Genzyme, and Merck Serono, Alexion, Chugai, MedImmune, Shire, Roche, Actelion, Celgene and serves on editorial Boards at PLos ONE (academic editor) and Neurology Neuroimmunology and Neuroinflammation (Associate Editor). He provided consultancies for SanofiGenzyme, BiogenIdec, MedImmune, Shire, Alexion; He received research support from Bayer, Novartis, Biogen Idec, Teva, Sanofi-Aventis/Genzyme, Alexion and Merck Serono, German Research Council (DFG Exc 257), Werth Stiftung of the City of Cologne, German Ministry of Education and Research (BMBF Competence Network Multiple Sclerosis), Arthur Arnstein Stiftung Berlin, EU FP7 Framework Program (combims.eu) Guthy Jackson Charitable Foundation, and National Multiple Sclerosis Society of the USA. JB-S has received travel grants and speaking honoraria from Bayer Healthcare, Biogen Idec, Merck Serono, Sanofi Genzyme, Teva Pharmaceuticals, Roche, and Novartis all unrelated to this work. AB is cofounder and shareholder of technology startups Motognosis GmbH and Nocturne GmbH. He is named as inventor on several patent applications describing serum biomarkers for multiple sclerosis, perceptive computing for motor symptoms and retinal image analysis using optical coherence tomography. HZ received research grants from Novartis and speaking fees from Bayer, unrelated to this study. The remaining authors declare that the research was conducted in the absence of any commercial or financial relationships that could be construed as a potential conflict of interest.
